# Metabolomics: The Key to Unraveling the Role of the Microbiome in Visceral Pain Neurotransmission

**DOI:** 10.3389/fnins.2022.917197

**Published:** 2022-06-23

**Authors:** Adam Shute, Dominique G. Bihan, Ian A. Lewis, Yasmin Nasser

**Affiliations:** ^1^Department of Medicine, Cumming School of Medicine, Snyder Institute for Chronic Diseases, University of Calgary, Calgary, AB, Canada; ^2^Department of Biological Sciences, University of Calgary, Calgary, AB, Canada

**Keywords:** visceral pain, inflammatory bowel disease, irritable bowel syndrome, microbiome, metabolomics

## Abstract

Inflammatory bowel disease (IBD), comprising Crohn’s disease and Ulcerative colitis, is a relapsing and remitting disease of the gastrointestinal tract, presenting with chronic inflammation, ulceration, gastrointestinal bleeding, and abdominal pain. Up to 80% of patients suffering from IBD experience acute pain, which dissipates when the underlying inflammation and tissue damage resolves. However, despite achieving endoscopic remission with no signs of ongoing intestinal inflammation or damage, 30–50% of IBD patients in remission experience chronic abdominal pain, suggesting altered sensory neuronal processing in this disorder. Furthermore, effective treatment for chronic pain is limited such that 5–25% of IBD outpatients are treated with narcotics, with associated morbidity and mortality. IBD patients commonly present with substantial alterations to the microbial community structure within the gastrointestinal tract, known as dysbiosis. The same is also true in irritable bowel syndrome (IBS), a chronic disorder characterized by altered bowel habits and abdominal pain, in the absence of inflammation. An emerging body of literature suggests that the gut microbiome plays an important role in visceral hypersensitivity. Specific microbial metabolites have an intimate relationship with host receptors that are highly expressed on host cell and neurons, suggesting that microbial metabolites play a key role in visceral hypersensitivity. In this review, we will discuss the techniques used to analysis the metabolome, current potential metabolite targets for visceral hypersensitivity, and discuss the current literature that evaluates the role of the post-inflammatory microbiota and metabolites in visceral hypersensitivity.

## Introduction

**FIGURE 1:**
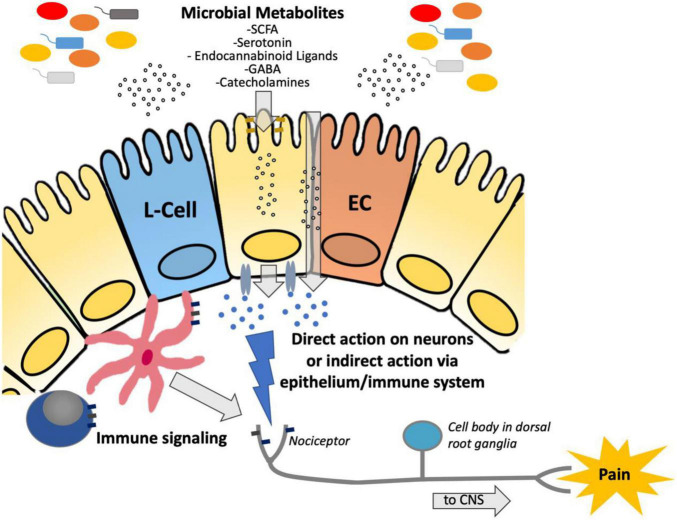
Diagram demonstrating the suggested pathway by which microbiota-derived metabolites are transferred across the epithelium to either (a) directly interact with nociceptors to modulate hypersensitivity or (b) indirectly act *via* immune stimulation to modulate hypersensitivity. SCFA, short chain fatty acids; GABA, Gamma-aminobutyric acid; ECC, enterochromaffin cell; CNS, central nervous system.

Inflammatory bowel diseases (IBD), including Crohn’s disease and ulcerative colitis (UC), as well as irritable bowel syndrome (IBS) are some of the most commonly diagnosed gastrointestinal disorders (Rakoff-Nahoum and Medzhitov, 2006). IBD are chronic debilitating illnesses, with increasing global incidence (Kaplan and Windsor, 2021). IBS is characterized by chronic abdominal pain associated with a change in bowel habits, affecting 11% of the population worldwide (Lacy et al., 2016). Both disorders have an associated high socioeconomic burden, poor quality of life and are associated with chronic abdominal pain (Piovani et al., 2020). The gut microbiome is known to affect a wide variety of gastrointestinal processes (Thursby and Juge, 2017) and plays a role in the pathogenesis of several gastrointestinal disorders, including IBD and IBS (Morgan et al., 2012; Pittayanon et al., 2019). Dysbiosis, or a change in the abundance and composition of bacteria, is characteristic of several gastrointestinal disorders, including IBD (Khan et al., 2019) and IBS (Pittayanon et al., 2019), although it is unknown whether these changes are causal to the disease or a consequence of changes in gastrointestinal motility, diet and gut inflammation. In humans with IBD and in animal models of colitis, sequencing of the intestinal microbiota (metagenomic or amplicon) has characterized phyla level shifts in the proportion of microbial species (Peterson et al., 2008; Tong et al., 2013). Whereas a healthy microbiota consists of the four major phyla, Firmicutes, Bacteroidetes, Proteobacteria, and Actinobacteria, this is often shifted in patients with IBD to a composition that is more abundant in Gram-negative species, such as Proteobacteria and Bacteroidetes (Peterson et al., 2008). As a result of these phyla level shifts, a decrease in overall species diversity within the colonic microbiome is commonly associated with IBD (Peterson et al., 2008). While these shifts in the composition of the gut microbiota have been extensively characterized in IBD, our understanding of the impact that these changes have on the intestinal metabolome is still developing. An emerging body of literature suggests that microbial metabolism plays a role in the pathogenesis of visceral hypersensitivity, through the production of neuroactive molecules such as neurotransmitters (Tsavkelova et al., 2000; Mawe and Hoffman, 2013; Pokusaeva et al., 2017) and microbial products of metabolism such as SCFA (Esquerre et al., 2020) (see Figure 1).

The metabolome refers to a collection of roughly 5,000 low molecular weight (<1 kD) molecules that are produced by microbes and host cells as a result of cellular metabolism (Bowling and Thomas, 2014). Metabolic processes play a fundamental role in all biological processes and an emerging body of literature suggests that host/microbiome dynamics can directly affect immune function (Mager et al., 2020; Yang and Cong, 2021), modulate the clinical presentation of diseases (Chu et al., 2019; Schirmer et al., 2019; Lavelle and Sokol, 2020), and may play a direct role in visceral pain. Although an emerging body of literature suggest that metabolism affects epithelial, neuronal, and immune function, the molecular mechanisms underlying these associations remain unclear. However, systematic interrogation of host/microbial dynamics using metabolomics approaches is proving new insights into how gut microbiota can modulate gastrointestinal diseases *via* metabolism.

One of the most common symptoms experienced by patients with IBD and IBS is abdominal pain (Brierley and Linden, 2014). Pain can be sub-divided into two sub-categories; visceral pain, which refers to the pain response originating within internal organs such as the intestine, while somatic pain refers to pain originating from muscle, bone, and soft tissue. In the context of IBD, 80% of patients report acute abdominal pain, which is associated with disease flares and increased intestinal inflammation and/or obstruction (Hurtado-Lorenzo et al., 2021). However, 30–50% of IBD patients experience chronic abdominal pain which can persist despite achieving endoscopic remission (Hurtado-Lorenzo et al., 2021). Individuals with IBD can also present with widespread somatic pain in the absence of inflammation (Regueiro et al., 2017), indicating altered sensory neural processing in this disorder. Most importantly, chronic abdominal pain in the absence of inflammation is a severe burden to patients, with significant associated anxiety, depression, and decreased in quality of life and increased healthcare utilization (Gracie et al., 2018). Studies investigating the pathophysiological mechanisms underlying chronic pain in IBD patients in remission are lacking, and effective treatments are just as limited (Hurtado-Lorenzo et al., 2021).

Chronic pain is a disorder of the brain gut axis, and both central and peripheral mechanisms contribute to its pathogenesis (Regueiro et al., 2017). Painful sensation from the gut is relayed to the central nervous system by nociceptors or pain-sensitive neurons with peripheral nerve terminals in the wall of the intestine (Brookes et al., 2013). Nociceptors have their cell bodies located in dorsal root ganglia and central terminal that synapse in the dorsal horn of the spinal cord; the colon is innervated by thoracolumbar and sacral afferents (Hockley et al., 2019), as well as vagal afferents (Borgmann et al., 2021; Jia et al., 2021). Nociceptors can be “sensitized,” defined as a decrease in the threshold for stimulation and an increase in the magnitude of the response, by neuropeptides and inflammatory mediators released by tissue damage (Gold and Gebhart, 2010). Nociceptor sensitization can lead to *hyperalgesia*, or an exaggerated pain response, as well as *allodynia*, or pain caused by what would be innocuous stimuli under normal conditions (Gold and Gebhart, 2010).

In both the clinical setting and in animal models, visceral pain can be assessed through the response to colorectal distention, where balloon distention of the colon is performed using a barostat to measure pain tolerance (Keszthelyi et al., 2012). In animal models, pain is measured quantitatively by either measuring the heart rate or the visceromotor response, which uses electrodes to measure abdominal contractions (Keszthelyi et al., 2012). Visceral hypersensitivity to colorectal distention is a hallmark pathophysiologic characteristic of chronic abdominal pain in IBD and IBS (Keszthelyi et al., 2012).

The current literature (Tsavkelova et al., 2000; Mawe and Hoffman, 2013; Pokusaeva et al., 2017; Esquerre et al., 2020) strongly implicates a role for the intestinal microbiota in the development of visceral hypersensitivity in the absence of active inflammation, suggesting that crosstalk between the host and the microbiota *via* microbial metabolites can result in visceral pain. Herein, we review the current state of the literature linking visceral pain to microbial metabolism and systematically review the proposed molecular mechanisms linking pain in the absence of active inflammation in IBD and IBS to microbial metabolism.

### Challenges in Studying Microbiome-Mediated Phenotypes

One of the primary challenges in studying the role that the microbiota plays in visceral pain is the significant logistical and biological complexities inherent to microbiome research. Microbes can have diverse metabolic capacities and the species-to-species differences in their ability to consume specific nutrients can have a profound impact on the metabolic composition of the gastrointestinal tract. Consequently, perturbations in the microbiome community can dramatically reshape the molecules that are ultimately passed along to the host. Although host- microbe interactions are known to play an important role in pain and many other physiological functions, unraveling this complexity is challenging in the context of microbiota studies.

Studying these metabolic phenomena is analytically challenging due to the broad chemical diversity of molecules that can potentiate host-microbe interactions. Short-chain fatty acids (SCFA) (Brestoff and Artis, 2013; Neis et al., 2015), amino acids (Wu et al., 2021), bile acids (Ridlon et al., 2014; Wahlström et al., 2016), hormones (Martin et al., 2019), secondary metabolites (Wang et al., 2019), and complex carbohydrates (Flint et al., 2012) are just a few examples of microbial metabolites that modulate host-metabolism. These broad chemical classes cannot all be captured on a single analytical platform and thus the selection of instrumentation can have a direct impact on biological mechanisms that can be investigated (see metabolomics platforms below).

Another factor which affects the complexity of microbiota profiling is that microbial community composition and its metabolome varies along the gastrointestinal tract (Kozik et al., 2019; Zhu et al., 2021). Thus, the microbial community composition is different between ileal luminal samples from colonic and fecal samples (Crespo-Piazuelo et al., 2018), as well along the length of the colon itself (Flynn et al., 2018). Within the colon, studies have demonstrated that fecal sampling does not fully capitulate the luminal microbial community of the proximal colon (Gu et al., 2013; Zhu et al., 2021). Furthermore, studies have demonstrated striking differences between luminal and mucosal samples within the colon itself, specifically regarding mucosa-associated bacteria such as *Bifidobacterium*, *Lactobacillus*, and *Akkermansia* (Heinsen et al., 2015; Kozik et al., 2019). In a study by Miyauchi et al. (2022) lavage samples were collected from 20 healthy donors, where the microbial community composition was compared to respective fecal samples. Substantial difference in microbial community composition within patients was observed between the two sample-types, with the lavage samples containing significantly more *Bifidobacteriaceae* and *Coriobacteriaceae* (Miyauchi et al., 2022). Given the substantial differences in the microbial community structure along the gastrointestinal tract, it can be expected the metabolome would also be different, thus adding to the challenges of mapping changes to the luminal metabolome. Furthermore, epithelial uptake of such metabolites along the intestinal tract would further impact the metabolomic composition within the fecal sample.

Another major challenge in studying microbial phenotypes is that the metabolic composition of the gastrointestinal tract and other sites results from the cumulative metabolic activity of the entire microbial community. Although individual microbes can produce unique molecules, most metabolites present in the gut can be consumed by a range of species. Moreover, the waste products generated through microbial metabolism can frequently be further metabolized by other gut microflora, thus creating complex chains of cross-feeding interactions that can obscure individual contributions to a host phenotype (Henriques et al., 2020).

An additional layer of complexity inherent to studying microbiome impacts on host physiology is that the metabolites produced by the gut microflora can have distal effects on a wide variety of host tissues. As has been shown in studies by Claus et al. (2008) and Wikoff et al. (2009), where the gut microflora has direct impact on the metabolic composition of peripheral tissues. Microbial metabolites are diffused or transported across the epithelium and can be detected throughout the body, such as in feces/intestinal biopsies (Rivera-Chávez et al., 2016), urine (Khamis et al., 2017), blood (Bi et al., 2020), liver (Raja et al., 2021), brain (Quintero et al., 2021), cerebral spinal fluid (Quintero et al., 2021), saliva (Chen and Yu, 2019), spleen (Jang et al., 2018), lymph fluid/nodes (Lee et al., 2019), muscle tissue (McGarrah et al., 2018), and lungs (Roque and Romero, 2021). Thus, unraveling pain phenotypes requires analysis of both the local and distal effects that could potentially arise from microbial metabolites.

### The Importance of Omics Research and Metabolomics in Evaluating the Microbiome

Over the last decade, analytical technology for tracking host-pathogen dynamics at the genomic, transcriptomic, proteomic, and metabolomics level has advanced considerably, and these tools now serve as the foundation for understanding complex phenotypes such as visceral pain (Moloney et al., 2016; Lucarini et al., 2022). Although whole genome sequencing of individual microbes remains an important element in understanding complex disease (Hollister et al., 2019; Minerbi et al., 2019), there has been an increasing shift toward using genomics to map microbial phylogenetic changes in the microbiome (Liu et al., 2021) *via* amplicon sequencing (16S rRNA) (Liu et al., 2021) or metagenomic (shotgun) sequencing (Peterson et al., 2008). Both of these approaches allow complex microbial communities to be mapped phylogenetically and can provide insights into how these populations change in response to complex human diseases, such as IBD (Amos et al., 2021). Though powerful, metagenomics is frequently limited in its ability to identify individual species and cannot distinguish between live and dead microbes. Consequently, this tool provides a definitive view of the overall phylogenetic composition of samples, but a limited view of biological activity (e.g., what proteins or metabolites are being secreted, and the host’s response to these molecules).

Limitations in genomics have been increasingly addressed *via* transcriptomic approaches, which can be used to map the mRNA, non-coding RNA, and micro-RNA present in the gut microenvironment. These studies are largely done through RNA sequencing (RNA-seq) strategies, which borrow heavily from metagenomics and are advantageous because they can capture transcripts from both host and microbe and thus can be used to map a comprehensive collection of genes that are activated or deactivated in response to IBD and other complex diseases (Lloyd-Price et al., 2019).

Proteomics approaches are also emerging as an important strategy for studying microbial environments (Aakko et al., 2020). Modern proteomics methods can quantify thousands of proteins in samples and recent advances in data independent acquisition (Aakko et al., 2020) and metaproteomics strategies (Kleiner, 2019) have dramatically improved the utility of this strategy for complex investigations of host/microbiome interactions. Proteomics strategies are particularly relevant in the context of researching metabolic mechanisms, as secreted enzymes (both from the host and the microbes) play a major role in catabolizing complex carbohydrates, lipids, proteins, and other nutritional sources from the gut and therefore have a direct impact on the composition of each microenvironment.

*Metabolomics* has emerged as a mainstream strategy for investigating metabolism on a systems-biology scale. Recent advances in mass spectrometry and informatics have made metabolomics much more accessible in recent years and these tools have been applied with increasing frequency to unravel complex host/microbiome metabolic interactions. A rapidly growing body of literature has shown that these intraspecies dynamics play a direct role in modulating the availability of nutrients (Claus et al., 2008; Wikoff et al., 2009; Koh et al., 2016; Putnam and Goodman, 2020), the pharmacokinetics of certain drugs (Taylor et al., 2019; Klünemann et al., 2021), and can modulate the clinical presentation of a wide range of diseases (Han et al., 2015; Blacher et al., 2019; Canfora et al., 2019; Mager et al., 2020). Importantly, several studies (Cai et al., 2022) have shown host/microbiome/metabolic connections may play a role in Parkinson’s disease (Mulak and Bonaz, 2015), depression (Painsipp et al., 2009), autism (Sharon et al., 2019), Alzheimer’s disease (Govindarajan et al., 2011), dementia (Liu et al., 2015), and a variety of other disorders that affect neuronal function (De Vadder et al., 2014; Strandwitz, 2018; Dalile et al., 2019). Although these microbial/host interactions appear to influence a wide spectrum of metabolic functions (e.g., amino acid, nucleotide, lipid, carbohydrate, vitamin/cofactors, and energy metabolism), much of the literature has centered on the role that SCFA and bile acids play in these complex diseases. However, this relatively narrow metabolic focus is beginning to broaden as a greater diversity of molecules are found to play a role in immunity (e.g., inosine Mager et al., 2020), dysbiosis (e.g., H_2_S; Sultan et al., 2021), and chronic disease (e.g., trimethylamine *N*-oxide; TMAO; Morgell et al., 2021).

Modern metabolomic studies are typically conducted using nuclear magnetic resonance (NMR) spectroscopy (Jacobs et al., 2008), gas chromatography mass spectrometry (GC-MS) (Hoving et al., 2018), and liquid chromatography mass spectrometry (LC-MS) (Chen et al., 2019). The relative merits, shortcomings, and pitfalls of each analytical platform has been extensively reviewed elsewhere (Lu et al., 2017). Briefly, GC-MS is most effective for analyzing volatile compounds and lipids, LC-MS is most effective for analyzing water-soluble compounds, and NMR is most appropriate when absolute quantification (Lewis et al., 2007; Markley et al., 2017) for unbiased detection of diverse molecular classes is paramount (Lewis et al., 2012).

Beyond these generalized characteristics, there are range of factors that affect the utility of each platform in the context of microbiome studies. Most importantly, the broad diversity of chemical classes involved in microbiome projects can significantly complicate the analytical strategy. SCFAs, for example, which are the subject of intense research in microbiome research, are most amenable to analysis by GC-MS (Zhang et al., 2019). Carbohydrates and alcohols, on the other hand, are most easily analyzed by NMR (Karu et al., 2018), while polar compound are most easily resolved by LC-MS using hydrophilic interaction liquid chromatography (HILIC) (Iturrospe et al., 2021). Consequently, no one technique can serve as a generalized platform for microbiome metabolomics. To address this, researchers either use a combination of techniques or will employ specialized analytical methods, generally involving chemical derivatization of the metabolites to improve their analytical properties for a particular platform. For example, aniline derivatization can be used to enable SCFA analysis by reverse phase (C18) LC-MS (Bihan et al., 2022), benzyl chloroformate can be used to improve the retention of amino acids *via* reverse phase chromatography (Peoples et al., 2018) and silylation can be used to improve the volatility of water-soluble analytes for analysis by GC-MS (Villas-Bôas et al., 2006). In summary, no single analytical platform is ideally suited to microbiome metabolomics and researchers must use combinations of techniques to capture the full breadth of chemical diversity inherent to this field. Despite these challenges, most project can be satisfactorily completed on either LC-MS or GC-MS platforms.

Although NMR is playing an increasingly smaller role in metabolomics due to its limited sensitivity (Markley et al., 2017; Karu et al., 2018), it still has important merits for microbiome analyses. One consideration is that NMR performance does not degrade over long cohorts. All MS-based platforms (especially LC-MS) suffer from progressive fouling of the electrospray source and ion optics that degrade instrument performance over time (Kang et al., 2017). In contrast, NMR samples are external to the electronics and thus are not subject to carry over or progressive fouling. This can help improve the reproducibility (Reade et al., 2014) of projects, especially in studies involving dirty samples (e.g., feces) which can lead to problems in MS analyses (Reade et al., 2014).

### Metabolomics and Microbial Community Dynamics

As expected, it is evident that alterations to microbial community diversity results in parallel changes in the metabolomic milieu of the intestinal microenvironment. Alterations to the intestinal microbiota resulting in changes to the metabolome occurs for a variety of reasons, such as dietary changes (Tang et al., 2019), antibiotic use (Theriot et al., 2014), disease states [i.e., IBD (Heinken et al., 2021), IBS (Mars et al., 2020), obesity (Cirulli et al., 2019), type 2 diabetes (Arneth et al., 2019)], host-genetics (Imhann et al., 2018), age (Houtkooper et al., 2011), breast feeding (Henrick et al., 2021), and mode of birth (Carter et al., 2019). With the rapid increase of sequencing technology over the last decade, studies highlighting the impact that these factors have on the intestinal microbiota is extensive, however, analysis on the impact that these alterations have on the metabolome are limited.

In the first 3-years of life, the host’s microbiota is developing rapidly to achieve a permanent state of homeostasis (Milani et al., 2017). During these first 3-years, alterations to the developing microbiota can result in life-long impacts on both the microbiome and metabolome (Milani et al., 2017). It is well known that microbes have diverse metabolic preferences. These differences have profound impacts on the complement of molecules that are taken up or secreted by the microbiota, which in turn changes both the community composition of the microbiota and host-metabolism (Le Chatelier et al., 2013; David et al., 2014). Studies have also demonstrated that the birth route, either vaginal or c-section, results in dramatic differences in the resident microbiota of the nasopharyngeal, skin, gut, and oral cavities (Shao et al., 2019). Babies that were delivered by c-section were found to have a microbiota that was dominated by *Staphylococcus* and *Streptococcus*, whereas vaginal delivery resulted in an increased abundance of *Lactobacillus* species (Marchioro et al., 2019). The differences in delivery methods were later shown to have profound changes to the infant metabolome. For example, cesarean-delivered babies had substantially lower glucose, inulin, non-esterified fatty acids, and acylcarnitine levels when compared to vaginal-delivered babies (Marchioro et al., 2019). Community differences within the intestinal microbiota is also observed between babies that were breast-fed and formula-fed, where breast-fed babies were found to have a greater abundance of species belonging to *Lactobacillus* and *Bifidobacterium* when compared to formula-fed infants (Phan et al., 2019). Combining the results of 12 studies that compared breast-fed and formula-fed infants, found a total of 261 fecal metabolites of which 151 were considered significantly different between the two feeding modalities. However, only 10 (acetate, alanine, creatine, glutamine, lactate, urea, citrate, formate, threonine, and glycine) of the 151 metabolites were consistently altered between the two feeding modalities (Phan et al., 2019). The majority of intestinal microbiome studies have been performed to evaluate the impact that diet has on both the microbiota and metabolome. A study by Schnorr et al. (2014), demonstrated that individuals who consumed a diet which was high in plant-derived fibers had a substantial increase in microbial community abundance in the stool, while in a later study, individuals who consumed a low-fiber diet were found to have lower intestinal microbial diversity (Sonnenburg et al., 2016). An increase in gut microbial diversity, *via* a high-fiber diet, also results in substantial increases in fermented metabolite products, such as SCFAs (Sonnenburg et al., 2016).

Antibiotics have a large impact on the intestinal microbiota, thus leading to substantial decreases in metabolites that are essential energy sources within the colon for colonocytes (Antunes et al., 2011). Several studies have demonstrated that treatment with antibiotics abolishes the overall community diversity within the intestinal tract (Antunes et al., 2011; Korpela and de Vos, 2016; Haak et al., 2019). The immediate and long-term impact that a 2-day treatment of broad-spectrum (ciprofloxacin, vancomycin, and metronidazole) antibiotics have on the intestinal microbiota is a decrease in species richness paired with a marked change in the community composition (Haak et al., 2019). Significant increases in Gram-negative bacteria, particularly proteobacteria, and decreases in obligate anaerobes were also seen after antibiotic treatment (Haak et al., 2019). Thirty one-months after broad-spectrum antibiotic treatment, there was partial but not full recovery in the diversity index (Haak et al., 2019). Thus, aggressive broad spectrum antibiotic treatment can have a long-lasting impact on the microbiota.

As expected, antibiotic treatment leads to equally long-term changes in the metabolome. Several studies have demonstrated the impact that antibiotic treatment has on the metabolome, where SCFAs and bile acids are commonly depleted in human and animal models (Theriot et al., 2014; Zhang Y. et al., 2014; Kuno et al., 2018; Zarrinpar et al., 2018). Clostridia represents one of the largest classes of anaerobic bacteria in the mammalian intestinal microbiota and are highly sensitive to antibiotics. Families within this class, particularly *Lachnospiraceae*, are well known butyrate-producing commensals (Theriot et al., 2014). This demonstrates the impact that aggressive antibiotic use has on not only the microbiota but on gut microbiota-derived metabolites.

As discussed earlier, environmental factors such as diet and antibiotic use have profound influences on the composition of the gut microbiota. Host genetics also has a significant impact on microbial composition. Genome-wide studies of both the host and the microbiota have identified variants in several human genes that are responsible for signaling, immunity, and epithelial-function which can in turn have a significant impact on overall gut microbial composition (Seregin et al., 2017; Imhann et al., 2018; Hu et al., 2021). Recent studies have also identified several genetic variants within individuals that can predispose a host to the onset of disease, such as IBS and IBD, and which are furthermore linked to increased visceral pain (Bonfiglio et al., 2021; Ledergerber et al., 2021; Vollert et al., 2022). A full discussion of these genetic alterations are beyond the scope of this review, however, a full discussion on host genetics and the microbiota in IBD was recently published by Qiu et al. (2022).

### The Microbiome and the Metabolome in Inflammatory Bowel Disease and Irritable Bowel Syndrome

#### Inflammatory Bowel Disease

One of the most explored gastrointestinal diseases when it comes to changes in microbial and metabolome composition in the last decade has been IBD. As described earlier, dysbiosis is common in patients suffering from IBD, where extensive community profiling studies describe phylum level shifts within the intestinal microenvironment of patients suffering from this disease. These studies, followed up with extensive targeted and untargeted metabolomics, found that patients with ulcerative colitis and Crohn’s lack microbiota-derived metabolites that are essential to maintaining proper gut health (Heinken et al., 2021). It remains unclear whether these changes to both the microbiota and metabolome are a cause or a result of intestinal inflammation. However, several studies demonstrate that these metabolomic changes are at the very least, preventing the host from achieving homeostasis within the intestinal tract (Lloyd-Price et al., 2019). A multi-omics study from the Human Microbiome Project which followed 132 individuals [consisting of patients with ulcerative colitis (UC), Crohn’s disease (CD), and healthy participants] for 1 year demonstrated that the fecal microbial community of UC patients with active disease was significantly different when compared to the control cohort (Lloyd-Price et al., 2019). UC patients demonstrated a significant reduction in community diversity and had a lower abundance of obligate anaerobes, specifically those belonging to the genus *Roseburia* and *Clostridium* clusters IV and XIVA. The effect of reduced anaerobes was evident in their metabolomic profiling, as UC patients had reduced levels of butyrate within their stool (Lloyd-Price et al., 2019).

Given the data that IBD-induced dysbiosis has a deleterious effect on the intestinal microenvironment, there have been several efforts to re-establish a “healthy” intestinal microenvironment in this disease. Although commonly used for the treatment of recurrent *C. difficile* infection, fecal microbiota transplant (FMT) is currently being explored as a preferred treatment option for active inflammation in UC and CD (Kelly C. R. et al., 2015). To date, studies have not demonstrated a significant benefit of FMT in either UC or CD, as only 28% of patients were able to achieve remission (compared to 9% in the placebo group), with 49% achieving a clinical response with treatment (compared to 28% in the placebo group) (Levy and Allegretti, 2019). In order to improve upon these lackluster findings, newer studies have incorporated prebiotic supplementation prior to FMT with some encouraging results (Scaldaferri et al., 2013). Additional studies with larger number of patients need to be performed to confirm these findings.

Another approach to restore the microbial microenvironment within the intestinal tract is through the use of pre- and/or probiotics. A prebiotic, such as high fiber supplementation, is a dietary nutrient that will enhance the growth of specific commensal bacteria and their metabolites in an effort to achieve intestinal homeostasis (Scaldaferri et al., 2013). In contrast, a probiotic is a single species or consortium of live strain specific bacteria that are cultured *in vitro* and are ingested by an individual to colonize the gastrointestinal tract (Scaldaferri et al., 2013). Probiotic treatment for active UC has been ongoing for several years, with the most commonly utilized strains being species within Clostridia, *Lactobacillus*, and *Bifidobacterium* (Jakubczyk et al., 2020). Various combinations of these microbes are currently in clinical trials, where their specific goal is to increase butyrate production within the colon in UC (Lavelle and Sokol, 2020). It is possible that these microbial-directed therapies will be used as adjuncts alongside biologic therapies in the induction and maintenance of remission in UC, although further studies are needed.

The positive impact that SCFAs have within the colon have been heavily characterized throughout the literature (Hinnebusch et al., 2002; Kim et al., 2014; Bachem et al., 2019). Butyrate is the primary energy source for colonocytes, but SCFAs have further positive effects beyond energy metabolism, such has decreasing luminal pH to enhance nutrient absorption (Lupton and Kurtz, 1993); maintaining microbiota composition *via* stimulating phagocytic activity (Wu et al., 2020); activating G-protein coupled receptors on neurons (Nøhr et al., 2015), epithelial (Al Mahri et al., 2020), enteroendocrine (Reimann and Gribble, 2016) and innate immune cells (Smith et al., 2013); inhibiting intracellular histone deacetylase activity (Hinnebusch et al., 2002); enhancing barrier function *via* tight junction protein stimulation (ZO-1 and Occludin) (Wang et al., 2018); stabilizing hypoxia-inducible factor (Kelly C. J. et al., 2015), and increasing Muc-2 mucin production (Gaudier et al., 2004). All these functions are pivotal in achieving homeostasis within the intestinal tract, primarily the colon.

It is clear that microbial metabolites play a significant contribution in limiting intestinal inflammation; in addition, the beneficial effects of metabolites, in particular SCFAs, can be observed systemically (Zaiss et al., 2015). Over 90% of SCFAs are absorbed by the colonic epithelium as a primary energy source for host cells, however, 10% are taken up by capillaries and transported *via* the portal vein to the liver prior to entering the systemic circulation (Topping and Clifton, 2001). Several systemic immune cells, epithelial cells and neurons express G protein coupled receptors for SCFAs including the free fatty acid receptor 2 (FFAR2), FFAR3, and G protein-like receptor 109A. Through these receptors, SCFAs are able to enhance metabolic activity and immune regulatory effects throughout the body (den Besten et al., 2013). The immune regulatory effects of SCFAs is an emerging field, and a full discussion of these findings can be found in a review by Parada Venegas et al. (2019).

#### Irritable Bowel Syndrome

IBS remains the most common reason for referral to gastroenterology and is associated with poor quality of life, anxiety, depression, and considerable economic burden (Ma et al., 2021; Shah et al., 2021). The pathogenesis of IBS is complex, with aberrant brain-gut interactions being at its center (Black and Ford, 2020). Several observations suggest that dysbiosis may play a key role in the pathophysiology of IBS. Patients can develop IBS after an episode of infectious enteritis, termed *post-infectious IBS*, with a range of bacterial pathogens including campylobacter, salmonella and shigella, being implicated. Both host and pathogen factors play a role in this process, with the severity of the illness and presence of elongating toxin being those associated with considerable relative risk (Barbara et al., 2016). Antibiotic therapy for non-enteric infections is also associated with an increased risk of developing IBS (Paula et al., 2015), suggesting that dysbiosis is an important risk factor in IBS pathogenesis. A recent systematic review demonstrated that there may be a “microbiome signature” in IBS, with an overall decrease in uncultured Clostridiales, in particular *Bifidobacterium* and *Faecalibacterium* genus, and an increase in *Lactobacillaceae*, Bacteroides, and *Enterobacteriaceae* when compared to controls, although there was considerable heterogeneity in the studies examined (Pittayanon et al., 2019). *Faecalibacterium*, in particular *F. prausnitzii*, are known to be anti-inflammatory, as well as major butyrate producers strongly associated with gut health (Lopez-Siles et al., 2017). Interestingly, *F. prausnitzii* was identified as a source of an anti-nociceptive serine protease that was able to decrease the excitability of mouse dorsal root ganglia neurons through a protease activating receptor -4 (PAR-4) dependent pathway (Sessenwein et al., 2017). It is tempting to speculate that a decrease in baseline *F. prausnitzii* may play a role in abdominal pain in IBS. However, given the correlative nature of these studies, it is difficult to know whether these microbial changes are causative to the disorder, or are secondary to changes in GI motility, diet, use of medications etc.

Given the link between symptom severity and the gut microbiome, it is not surprising that therapies targeting dysbiosis are being used to treat IBS. A recent systematic review and meta-analysis identified that probiotics, in particular multi-strain formulations, have a modest effect on IBS symptom severity (Ford et al., 2018) but the mechanism of action for the most part remains unclear. There is some speculation that probiotics and bacterial metabolites can signal directly to the brain to improve central symptoms associated with IBS (termed “psychobiotics,” reviewed in Sarkar et al., 2016). A recent randomized controlled trial of 44 adults with IBS with diarrhea-predominance or mixed bowel habits and mild to moderate anxiety and/or depression compared treatment with the probiotic *Bifidobacterium longum* NCC3001 to placebo (Pinto-Sanchez et al., 2017). Patients treated with *B. longum* showed a significant reduction in depression scores and an associated improvement in quality of life, with functional MRI studies showing reduced responses in regions of the brain that process negative emotion. Evaluation of urine metabolomics demonstrated an increase in methylamines and aromatic amino acids, including the host-bacterial co-metabolite 4-cresol sulfate, decreased levels of which are associated with depression. Interestingly, no change in IBS symptom severity or fecal microbiota profiles were seen in *B. longum*-treated patients, suggesting direct signaling of *B. longum* metabolites to the central nervous system (Pinto-Sanchez et al., 2017). These data suggest that probiotics may play a complex role in the treatment of IBS.

Other strategies to normalize the intestinal microenvironment including antibiotics, FMT and prebiotics/dietary modification have also been used to treat IBS. The non-absorbable antibiotic rifaximin is approved for the treatment of diarrhea-predominant IBS patients and has modest effects on IBS symptom severity (Pimentel et al., 2011; Lembo et al., 2016). However, the mechanism of action of rifaximin is unclear, with only minimal changes in the composition of gut microbiota being observed (Ford et al., 2018; Pimentel and Lembo, 2020). FMT has been evaluated in randomized controlled trials in IBS, but a recent systematic review and meta-analysis did not demonstrate significant improvements in symptom severity when compared to placebo, although there was some heterogeneity depending on the modality of FMT delivery (Ianiro et al., 2019).

Perhaps the best studied microbial strategy to treat IBS is dietary modification. Patients often associate gut symptoms with food consumption and show a preference toward dietary treatment (Sturkenboom et al., 2022). The low FODMAP (fermentable oligo- di- mono-saccharide and polyol) diet has gained considerable traction to treat IBS and is superior to other dietary interventions (Black et al., 2021). FODMAPs are fermentable prebiotics, which are thought to increase colonic gas production, causing visceral hypersensitivity to colonic distention in IBS patients (Major et al., 2017). Interestingly, the low FODMAPs diet has also been shown to be effective for abdominal pain in IBD patients in remission (Prince et al., 2016; Cox et al., 2020), suggesting that “saccharolytic-rich dysbiosis,” may be a common microbial cause that contributes to the pathogenesis of visceral hypersensitivity in both IBD and IBS patients (Chumpitazi et al., 2015; Rossi et al., 2018).

Metabolomics can be used to predict the response to the low FODMAPs diet. The presence of certain volatile organic compounds in fecal samples was able to discriminate responders from non-responders in a randomized cross-over trial of IBS patients (Rossi et al., 2018). Another study that randomized IBS patients to either a low or high FODMAPs diet found significant differences in urine metabolite profiles after the 3-week study. Amongst the altered metabolites was histamine, which was elevated at baseline and was significantly decreased after the low FODMAPs diet (McIntosh et al., 2017). The source of histamine may be either host- or microbial-derived; histamine is known to participate in the pathogenesis of visceral hypersensitivity *via* sensitization of nociceptors through the histamine-1 receptor (De Palma and Bercik, 2022). These data suggest that metabolomics may allow the identification of patients who will benefit from this type of treatment strategy.

There are concerns, however, regarding the long-term use of this restrictive diet in IBS, as the low FODMAPs diet leads to a decrease in SCFA-producing species, such as *Bifidobacteria* (Halmos et al., 2015). Even after dietician guidance and careful reintroduction, fecal SCFA content remains decreased despite a more “personalized” FODMAPs restriction, the long-term consequences of which are not known. However, this “personalized” approach results in long term symptom improvement and patient satisfaction in IBS (Staudacher et al., 2022). Interestingly, there are data that suggest SCFAs may be involved in the pathogenesis of visceral hypersensitivity, as discussed below.

### Microbial Metabolites Play a Role in Visceral Pain

Although several studies have demonstrated the beneficial impact that SCFAs have on host metabolic function and immune regulation, studies investigating the effect that these and other microbial metabolites have on the nervous system, specifically with regards to pain, are still emerging. Microbes can produce neuroactive molecules, such as toxins (Chiu et al., 2013; Blake et al., 2018; Yang et al., 2021), neurotransmitters (Tsavkelova et al., 2000; Mawe and Hoffman, 2013; Pokusaeva et al., 2017), proteases [which stimulate neuronal protease-activated receptors (PAR)] (Sessenwein et al., 2017), and metabolites including SCFAs (Baj et al., 2019; Lomax et al., 2019). Bacterial products can signal directly to nerves, or can act indirectly through the immune system, epithelial cells, or enteroendocrine cells to activate nociceptors (Lagomarsino et al., 2021) (see Figure 1). Both vagal (Borgmann et al., 2021; Jia et al., 2021) and spinal afferents are involved in nociception.

The gut-brain axis is a bidirectional signaling pathway between the central nervous system and the gut (Lomax et al., 2019). Indeed, several studies have shown that neurological diseases can alter the gut microbiota (Quigley, 2017), while a dysbiotic microbiota has also been shown to change behavior (Pinto-Sanchez et al., 2017). This data highlights a key role of the microbiota in the gut-brain axis, in which bacterial metabolites play a crucial role in this bidirectional communication. As described below, there are several studies that have shown a role for the microbiota in the pathogenesis of visceral hypersensitivity, however, studies exploring the role of the metabolome are limited. Thus far, there have been a handful of studies characterizing metabolite interactions with the nervous system.

Previous studies using germ-free models (Luczynski et al., 2016) and antibiotic-treated (Verdú et al., 2006; Hoban et al., 2016) models have shown that the microbiota plays a role in visceral pain. Germ-free mice are mice that were breed under sterile conditions and remain sterile their entire life; their intestinal tract lacks a microbiota (Bhattarai and Kashyap, 2016). Antibiotic treatment depletes and alters the intestinal microbiota. It is important to note that antibiotic-treated and germ-free models each have their own advantages and disadvantages. Germ-free mice are considered immunocompromised with distinct physiological and metabolic deficits (Bhattarai and Kashyap, 2016); these animals also demonstrate an altered enteric nervous system (Collins et al., 2014; Filipe et al., 2018). Antibiotic-treated mice have a competent immune system and GI physiology that is unaltered from naïve mice, however, antibiotics can have off-target effects, including causing low-grade intestinal inflammation, as well as increasing visceral hypersensitivity alone (O’Mahony et al., 2014). A study by Vicentini et al. (2021) demonstrated that broad spectrum antibiotic treatment in mice affected the structure and function of the GI tract, resulting in a loss of enteric neurons in both the submucosal and myenteric plexuses. However, supplementation with SCFAs post-antibiotic treatment restored enteric neuronal loss (Vicentini et al., 2021).

Using a germ-free model, Luczynski et al. (2016) demonstrated that male mice had increased visceral hypersensitivity to colorectal distention when compared to their specific pathogen free (SPF) littermates. Furthermore, recolonization could reverse this visceral hypersensitivity. Interestingly, germ-free females did not have increased visceral hypersensitivity when compared to SPF counterparts; estrous-cycle induced changes in visceral pain were abolished in the germ-free animals (Tramullas et al., 2021). Visceral hypersensitivity to colorectal distention was also observed in mice treated with broad-spectrum antibiotics; this change was accompanied by increases in substance P immunoreactivity (Verdú et al., 2006). In another study, the fecal microbiota transplant model was used to evaluate the role of the microbiota in visceral hypersensitivity. Crouzet et al. (2013) colonized gnotobiotic rats with a microbiota that replicated IBS dysbiosis (consisting of more sulfate-reducing bacteria and *Enterobacteriaceae* and less *Bifidobacteria*). It was found that the rats who received IBS-like microbiota had increased visceral hypersensitivity when compared to gnotobiotic rats that received a healthy microbiota, suggesting that IBS-associated hypersensitivity is in part caused by changes to the intestinal microbiota (Crouzet et al., 2013; De Palma et al., 2017).

### Metabolites and Visceral Pain

See Table 1 for a simplified description of studies demonstrating the role and mechanism of metabolites in visceral pain.

**TABLE 1:** Role of microbial metabolites in visceral pain.

Metabolite	Role and mechanism	Model system	References
SCFA	Direct sensitization of TRPV1 expressing nociceptors, increases visceral hypersensitivity *via* a MAP-K dependent pathways	Cultured mouse nociceptors; post-inflammatory DSS mouse; rat model	Xu et al., 2013; Esquerre et al., 2020
	Butyrate enemas decrease visceral hypersensitivity—mechanism not defined	Healthy patients, mice, rats	Tarrerias et al., 2002; Vanhoutvin et al., 2009; Russo et al., 2016
	Indirect mechanism whereby SCFA stimulate L-cells to release GLP-1 which reduces visceral hypersensitivity	Mouse model and mixed colonic cell culture	Gong et al., 2014; Psichas et al., 2015
	Indirect mechanism—SCFA induces 5HT release from EC which can then increase visceral hypersensitivity	Germ-free mouse, Human EC cell line	Reigstad et al., 2015
DCA	Direct increase in excitability of nociceptors	Mouse model; cultured mouse nociceptors	Yu et al., 2019
	Indirect increase in nociceptor excitability *via* 5HT3 dependent release	Mouse model	Yu et al., 2019
	Indirect increase in visceral pain *via* NGF release from mast cells	Rat model	Li et al., 2019
GABA	Synthesized by *Lactobacillus* and *Bifidobacterium* species and decreases visceral hypersensitivity	Rat fecal retention model, Mouse model	Hara et al., 1999; Pokusaeva et al., 2017

*Metabolites which play a putative role in pain neurotransmission (e.g., microbial-derived endocannabinoids, tryptophan metabolites, catecholamines) are not included. For a full discussion see pages 26–33. SCFA, Short Chain Fatty Acids; TRPV1, transient receptor potential vanilloid-1; DCA, deoxycholic acid; NGF, nerve growth factor; EC, enterochromaffin cells.*

#### Short-Chain Fatty Acids

The post-inflammatory dextran sodium sulfate (DSS) colitis model is an established model of chronic visceral pain, mimicking chronic pain in the post-inflammatory state in IBD. Mice are allowed to recover for 5 weeks after exposure to chemically induced colitis, and then develop visceral hypersensitivity to colorectal distention (Esquerre et al., 2020). Esquerre et al. (2020) demonstrated that FMT of post-inflammatory DSS stool into antibiotic-treated mice resulted in visceral hyperalgesia compared to antibiotic treatment alone; FMT of control stool dampened visceral hypersensitivity (Esquerre et al., 2020). Post-inflammatory mice exhibited changes in the microbiome, with significant increases in SCFA-producing species, such as *Lachnospiraceae* and *Ruminococcus*, and stool SCFA content when compared to control mice. Importantly, SCFAs were able to sensitize transient receptor potential vanilloid-1 (TRPV1) expressing nociceptors, suggesting that microbial-derived metabolites play a role in post-inflammatory pain (Esquerre et al., 2020).

Esquerre et al. (2020) showed a pro-nociceptive effect of SCFA, which is at odds with the ability of SCFAs to reduce colonic inflammation and immune activation. Interestingly, another study investigated the ability of SCFA enemas to improve visceral hypersensitivity in a haptenizing model of colitis, 2,4,6-trinitrobenzenesulfonic acid solution (TNBS), in rats (Tarrerias et al., 2002). Although visceral hypersensitivity was reduced in control rats that received butyrate enemas, visceral pain remained unchanged in rats that were exposed to TNBS and treated with butyrate enemas (Tarrerias et al., 2002). In healthy patients and mice, butyrate enemas caused a reduction in abdominal pain to colorectal distention (Vanhoutvin et al., 2009; Russo et al., 2016). In contrast, butyrate enemas increased visceral hypersensitivity through a MAP kinase dependent pathway in rats (Xu et al., 2013).

SCFAs can also modulate visceral hypersensitivity through an indirect mechanism. The SCFA receptors, FFAR2 and FFAR3 are highly expressed on intestinal L cells which contain GLP-1 (Chimerel et al., 2014). When stimulated by SCFAs, L-cells release glucagon like-peptide-1 (GLP-1) (Psichas et al., 2015). This increased secretion was not observed in mice lacking FFAR2 or FFAR3 (Psichas et al., 2015). Activation of the GLP-1 receptor on neurons can reduce visceral hypersensitivity (Gong et al., 2014). Taken together, these data show that SCFAs play a role in visceral hypersensitivity, but further studies are needed to understand the mechanism behind this and to reconcile data showing both pro- vs. anti-nociceptive roles.

#### Bile Acids

Bile acids are thought to play a role in the pathogenesis of IBS as a subset of patients with diarrhea-predominant IBS have an increase in colonic bile acids and can be treated with bile acid sequestrants (Wadhwa et al., 2015). Bile acids are traditionally associated with their role in lipid absorption. Primary bile acids are synthesized by the liver and undergo deconjugation by colonic bacteria to form multiple secondary bile acids, including deoxycholic acid (DCA; recently reviewed; Ní Dhonnabháín et al., 2021). DCA may play a role in visceral hypersensitivity. In a mouse model, DCA was able to increase afferent nerve firing by stimulating 5HT release from EC cells. In the proximal colon, the effect of DCA was inhibited by a 5HT3 receptor antagonist. However, DCA was also able to increase the excitability of nociceptors directly, in a 5HT-independent manner (Yu et al., 2019). In a separate study, colonic DCA enemas increased visceral pain to colonic distention in a rat model, an effect which involved the release of mast cell-derived nerve growth factor (NGF) (Li et al., 2019). NGF was able to increase the expression of neuronal TRPV1, a key receptor involved in nociception. Mast cells are known to form close contacts with nerve terminals in seminal biopsy studies of IBS patients and participate in the pathogenesis of visceral hypersensitivity (Barbara et al., 2007, 2004; Hasler et al., 2022). Interestingly, studies report an increase in secondary bile acids in diarrhea-predominant IBS, due to an excess of *Clostridia-*rich microbiota in this disease (recently reviewed; Gu et al., 2022). Thus, it is possible that secondary-bile acid induced visceral hypersensitivity contributes to the pathogenesis of abdominal pain *in vivo*.

#### Serotonin and Tryptophan Metabolism

Serotonin is a major neurotransmitter within the gastrointestinal tract, that plays an essential role in GI motility. Indeed, drugs targeting the serotonin receptor 5HT3, which is expressed on nociceptors, have been extensively studied for the treatment of visceral hypersensitivity (Mawe and Hoffman, 2013). Serotonin also plays a key role in microbial sensing *via* enterochromaffin (EC) cells, which are specialized neuroendocrine cells lining the intestinal epithelium that are responsible for GI motility and enzyme secretion (Mawe and Hoffman, 2013; Legan et al., 2022). A study by Reigstad et al. (2015) demonstrated that the rate limiting enzyme for serotonin synthesis, tryptophan hydroxylase (TH) was increased in germ-free mice colonized with human stool compared to germ-free mice alone. *In vitro* treatment of a human EC cell line with SCFA increased TH production (Reigstad et al., 2015). This data demonstrates that bacterial-derived luminal SCFAs can be detected by EC cells, which in turn secrete basolateral serotonin when activated. A study performed by El-Ayache and Galligan (2019), determined that disrupting the serotonin reuptake transporter (SERT) in female rats increased visceral hypersensitivity, through increased serotonin signaling at dorsal spinal 5HT3 receptors. However, the same phenomenon was not seen in male rats (El-Ayache and Galligan, 2019), suggesting a sex-dependent pain pathway which has been previously reported before in the CNS (Kogler et al., 2016; Mapplebeck et al., 2017). Although these studies do not show that the microbiota directly cause visceral hypersensitivity, there is clear evidence to suggest that communication between the microbiota and the host facilitates visceral hypersensitivity.

Dietary tryptophan is metabolized to 5HT in EC cells but is a substrate for the kynurenine pathway in the epithelium and immune cells, and the indole pathway in gut microbes. Indole derivatives bind to the aryl hydrocarbon (AhR) receptor (Agus et al., 2018). Dysbiosis and a subsequent alteration in tryptophan metabolism is thought to contribute to the pathogenesis of several GI diseases, including IBD and IBS (Kennedy et al., 2017; Agus et al., 2018). Peripheral kynurenine activity was shown to be correlated with the severity of IBS symptoms (Fitzgerald et al., 2008). In an animal model of IBS, decreased activity of the indole pathway and AhR-dependent IL-22 production, was correlated with anxiety-like behaviors; visceral pain was not evaluated in this report (Maëva et al., 2022). However, dysregulated tryptophan metabolism may contribute to the pathogenesis of visceral pain through altered central serotonergic functioning, and subsequent changes in central pain perception (Labus et al., 2011). Thus, microbial tryptophan metabolism may modulate 5HT-dependent visceral pain through both central and peripheral pathways.

#### Gamma-Aminobutyric Acid

The microbiota has the capacity to synthesize and secrete functional neurotransmitters. Gamma-aminobutyric acid (GABA), it is the main neurotransmitter within the central cortex and spinal cord (Pokusaeva et al., 2017). Species belonging to *Lactobacillus* and *Bifidobacterium* have been identified as a source of intestinal GABA production (Yunes et al., 2016). In rats, agonists of GABA receptors have demonstrated the ability to inhibit colorectal distention induced visceral pain (Hara et al., 1999). In a study by Pokusaeva et al. (2017), *Bifidobacterium dentium* was shown to produce GABA *via* enzymatic decarboxylation of glutamine. Probiotic supplementation of *Bifidobacterium* suppressed neuronal activity resulting in reduced visceral hypersensitivity in a rat fecal retention model (Pokusaeva et al., 2017). Thus, signaling through GABA receptors *via* microbial-derived GABA can prevent visceral hypersensitivity. Interestingly, analysis of the fecal metabolome in individuals suffering from IBD as well as IBS demonstrated a depletion in GABA levels (Aggarwal et al., 2018; Heinken et al., 2021), suggesting that this may represent a key mechanism whereby the dysbiotic microbiota can modulate visceral pain.

#### Catecholamines

Catecholamines are monoamine neurotransmitters or hormones used to induce stimulation and response within the mammalian body. There are three main catecholamines: epinephrine, norepinephrine, and dopamine. Dopamine is the major neurotransmitter involved in reward-motivation behavior and is a precursor for the other catecholamines: norepinephrine and epinephrine. Norepinephrine and epinephrine are responsible for the “fight or flight” response. The release of norepinephrine in response to heterotypic chronic stress in rats demonstrated a direct role for this neurotransmitter in increasing visceral hypersensitivity to colorectal distention. Norepinephrine was able to increase the expression of nerve growth factor along the colonic epithelium, which was then able to sensitize nociceptive nerves in the absence of inflammation (Winston et al., 2010). Commensal bacteria that reside within the microbiota have demonstrated the ability to respond to and produce these catecholamines. A study by Freestone et al. (2002) demonstrated that the growth rate of pathogenic enterohemorrhagic *E. coli* was increased in the presence of norepinephrine and dopamine. This effect was commonly observed in other pathogenic bacteria as well (O’Donnell et al., 2006). Several commensal bacteria, particularly *Bacillus* sp., have demonstrated the ability to produce norepinephrine and dopamine (Tsavkelova et al., 2000). Thus, it is possible that microbial-produced neurotransmitters may play a role in the pathogenesis of visceral pain.

#### Endocannabinoids

Cannabinoids have long been used to treat abdominal pain and disorders of GI motility (Izzo and Sharkey, 2010; Goyal et al., 2017) and are extensively utilized by patients with IBS and IBD (Adejumo et al., 2019; Nasser et al., 2020; Bogale et al., 2021; Hryhorowicz et al., 2021). There is evidence that the body’s endogenous cannabinoid system, the endocannabinoid system, which is involved in the control of gastrointestinal motility, sensation and visceral pain, is altered in both IBS (Camilleri et al., 2008, 2013; Fichna et al., 2013; Zhang S.-C. et al., 2014) and IBD (Storr et al., 2009, 2010; Alhouayek and Muccioli, 2012; Strisciuglio et al., 2018). Interestingly, the gut microbiome interacts with the endocannabinoid system (Hosseinkhani et al., 2021; Iannotti and Di Marzo, 2021), while endocannabinoids have been shown to modulate microbiota-driven changes in pain neurotransmission (Rousseaux et al., 2007; Aguilera et al., 2013; Cani et al., 2016; Rea et al., 2021). For example, the probiotic *Lactobacillus acidophilus* was able to induce the expression of the cannabinoid CB2 receptor as well as the μ-opiate receptor in epithelial cells both *in vitro* as well as *in vivo* in rodent models, which in turn led to a decrease in visceral sensitivity (Rousseaux et al., 2007). Commensal bacteria can produce endocannabinoid-like molecules, such as the anandamide-like N-acyl amides (Cohen et al., 2017) and the linoleic acid metabolite 10-oxo-12(*Z*)-octadecenoic acid (Kim et al., 2017). 5HT3 receptor-dependent release of anandamide in the duodenum is known to be anti-nociceptive (Feng et al., 2014) in a rat model, while linoleic acid metabolites have been reported to sensitize TRPV1, and increase both mechanical and thermal hypersensitivity (Sisignano et al., 2016). It remains to be determined whether a microbial source of endocannabinoid-like molecules plays a role in visceral hypersensitivity.

### Vagal Afferent Stimulation by the Microbiota

Recently, there has been exciting data indicating that vagal afferents may be involved in nociception. Vagal afferents are known to modify central pain processing in the spinal cord and brain (Bonaz et al., 2016). Vagal afferents express TRPV1 (Dworsky-Fried et al., 2020), SCFA receptors FFAR3 (Nøhr et al., 2015), as well as TLR4 (Jia et al., 2021), suggesting that microbial metabolites released within the gastrointestinal tract can modulate visceral pain within the host. In a recent study by Jia et al. (2021), it was demonstrated that lipopolysaccharide (LPS) was able to activate TLR4 on vagal afferents, which stimulated the release of calcitonin gene-related peptide (CGRP) release from vagal ganglia. They found that Tlr4 mRNA was enriched in vagal afferents expressing the sodium channel Nav1.8, which is well known to play a role in pain neurotransmission (Nguyen and Yarov-Yarovoy, 2022). These afferents also co-expressed CGRP (Jia et al., 2021). Although this particular study did not evaluate visceral pain, it is well known that CGRP signaling may be involved in afferent nerve sensitization and visceral organ hypersensitivity (Plourde et al., 1997; Delafoy et al., 2006; Noor-Mohammadi et al., 2021). Patients with IBD and IBS are reported to have a decrease in vagal tone (Pellissier et al., 2010). Subdiaphragmatic vagotomy as well as the application of lidocaine to abdominal vagal nerves was shown to blunt the response to colorectal distention, suggesting a potential anti-nociceptive role of vagal afferent stimulation (Chen et al., 2008). For a full discussion on this topic, the reader is referred to an excellent recent review on vagal/microbial interactions that was recently published by Bonaz et al. (2018).

## Conclusion and Future Directions

The staggering increase in IBD diagnoses each year across the developed regions of the world has been a large focus of research and drug development, as there is a dire need for new therapies with limited side effects. With this rapid increase in cases, patients that achieve endoscopic remission have persistent abdominal pain and visceral hypersensitivity. Studies to date have shown that the microbiota are involved in the pathogenesis of visceral hypersensitivity. However, the majority of these are strictly observational, where germ-free or antibiotic models are paired with amplicon sequencing to characterize a role for the microbiota in visceral hypersensitivity. Few studies have evaluated changes to the human metabolome in patients with visceral hypersensitivity, with even fewer studies taking these observed metabolomic changes and evaluating the interaction that these metabolites have on the host pain response in both the periphery and the central nervous system. In the last decade, the field of metabolomics has made great advancements, and the current techniques of targeted and untargeted analysis of a heterogenous samples (such as the feces or biopsies) can be utilized to identify specific metabolites unique to patients suffering from visceral hypersensitivity. These can in turn be tested in animal models and *in vitro* systems to evaluate putative mechanisms underlying visceral pain and hypersensitivity. This may in turn lead to future targeted treatments for visceral pain, either through the use of FMT, pro/prebiotics, dietary therapies, targeted antibiotics, or metabolite receptor- agonists/antagonists. Future studies need to move away from current observational based community profiling experiments and investigate direct and indirect mechanisms whereby microbial metabolites sensitize nociceptors.

## Author Contributions

AS primarily drafted the manuscript. DB, IL, and YN drafted portions of the manuscript and critically revised the manuscript for important intellectual content. All authors approved the final version for submission.

## Conflict of Interest

The authors declare that the research was conducted in the absence of any commercial or financial relationships that could be construed as a potential conflict of interest.

## Publisher’s Note

All claims expressed in this article are solely those of the authors and do not necessarily represent those of their affiliated organizations, or those of the publisher, the editors and the reviewers. Any product that may be evaluated in this article, or claim that may be made by its manufacturer, is not guaranteed or endorsed by the publisher.
